# Ultrathin S-Band Multifunctional Metamaterial with Broadband Microwave Absorption and Hydrophobic Characteristics

**DOI:** 10.3390/nano16100620

**Published:** 2026-05-18

**Authors:** Hongxu Jin, Huifang Pang, Renguo Guan, Siqi Yin, Wang An, Changfeng Wang

**Affiliations:** Engineering Research Center of Continuous Extrusion, Ministry of Education, Dalian Jiaotong University, Dalian 116028, China

**Keywords:** multifunctional metamaterial, S-Band broadband absorption, ultrathin thickness, hydrophobicity

## Abstract

Effective absorption in the S-band usually requires relatively thick absorbing materials. However, growing application demands necessitate the development of high-performance materials with subwavelength thickness. This study presents a broadband absorbing metamaterial for the S-band, based on a novel structural design featuring a nested hexagonal metal resonant layer integrated with a carbonyl iron powder (CIP)/charcoal (CH)/epoxy resin (ER) composite slab. This structural innovation enables exceptional S-band absorption within a subwavelength thickness, effectively overcoming the inherent physical limitations of traditional materials. By combining the arch measurement method and simulations over the 2–18 GHz, we demonstrate that the metal resonant layer of the metamaterial plays a key role in controlling the electromagnetic field vector distribution. This work investigates the mechanism for enhancing S-band absorption in metamaterials through the redistribution of electromagnetic field vectors. Additionally, magnetic loss from CIP/CH/ER and dielectric loss from the resonators further enhance absorption performance. The designed absorbing metamaterial exhibits effective absorption at a thickness of only 2.25 mm, with a reflection loss (RL) below −10 dB from 2.2 to 3.8 GHz. Simultaneously, it can maintain a radar cross-section (RCS) below −10 dBm^2^ in a wide-angle range of ±160°. Furthermore, a superhydrophobic coating with a contact angle of 152° was prepared for absorbing metamaterial. This coating allowed the metamaterial to preserve its microwave absorption performance while imparting self-cleaning capability. This study proposes a multifunctional absorbing metamaterial for efficient absorption in the S-band.

## 1. Introduction

The rapid development of wireless technology has led to the expanding application of the S-band (2–4 GHz) in fields like communications, radar detection, and aerospace, while also giving rise to serious concerns about electromagnetic pollution. This pollution can impair the operation of electronic devices and threaten human health. Consequently, developing high-performance absorbing materials has become critically important for both defense and civilian industrial applications. Traditional absorbing materials are constrained by the Planck–Rozanov limit [1,2], which makes it difficult to achieve high absorption efficiency while maintaining a lightweight structure. Common broadband designs that rely on increased thickness inevitably have excessive weight, limiting their suitability for most real-world applications.

Metamaterials [3,4,5,6,7] are artificially engineered composites with unique structural designs that contain extraordinary electromagnetic properties surpassing those of natural materials. Such materials have shown significant bandwidth enhancements in absorption through innovative architectural configurations. For instance, Liu et al. [8] reported a prism–honeycomb nested structure achieving over 90% absorption (RL < −10 dB) across 1–18 GHz with a thickness of only 7 mm. Similarly, Sun et al. [9] developed a honeycomb-based metamaterial incorporating aramid nanofibers and MXene nanosheets, attaining near-complete X-band coverage (RL < −10 dB) at a minimal thickness of 1.9 mm. Huang et al. [10] demonstrated a chiral metamaterial based on CIP that achieved an effective bandwidth of 7.7–15.88 GHz at a structural height of 2.5 mm. Beyond single-layer designs, the multi-layer metamaterials fabricated by combining high-loss-absorbing materials with metal resonant array structures exhibit excellent absorbing properties [11,12]. Du et al. [13] designed a structure with a top layer of patterned split rings and metal strips, achieving effective absorption from 1.24 to 3.14 GHz at a 9 mm thickness. Shou et al. [14] dutilized magnetic layers and an etched FR4 substrate to cover the 1.73–4.04 GHz range within a 3.4 mm. Although efficient absorption of different frequency bands has been achieved at various thicknesses through specific structural designs, realizing S-band broadband absorption at a thickness of 3 mm remains a major challenge.

Due to the severe conditions of absorbing materials, their restricted durability significantly hampers both their functionality and real-world utility. Recent studies [15,16,17,18] indicate that hydrophobicity is primarily achieved through the design of surface microstructures in absorbing materials. Que et al. [19] fabricated fluorinated graphene@copper hybrids featuring multi-interfacial heterostructures that demonstrate excellent superhydrophobicity with a water contact angle (WCA) of 154.0°. Tian et al. [20] employ a simple one-pot synthesis method to prepare fluorine-free core–shell CIP, achieving hydrophobicity with a WCA of 132.5°. Microstructural design enables hydrophobicity in absorbents, yet complex processing and high costs limit their large-scale application in engineering. The high-fluidity epoxy matrices may encapsulate modified material surfaces during coating preparation, drastically compromising hydrophobicity. Therefore, designing coatings that combine superior hydrophobicity with suitability for large-scale production remains a challenge for engineering applications.

In this study, we fabricated broadband S-band metamaterial absorbers by combining CIP/CH/ER composite with nested hexagonal array metallic resonant layers. We investigated the RL characteristics by adjusting the CIP/CH weight ratio and optimizing the metallic resonant layer design. This revealed the synergistic effect between material composition and structure in enhancing microwave absorption. The developed metamaterial demonstrates broad absorption bandwidth across the S-band. We employed finite-element simulations to systematically analyze the attenuation mechanisms of absorbing metamaterials. Specifically, we elucidated how the metallic resonant layer influences the electromagnetic field to enhance microwave loss. RCS simulations confirm the excellent radar wave attenuation of the metamaterial across a wide incidence angle range. For practicality, a superhydrophobic coating was designed for the metamaterial, which bolsters durability in harsh environments through its self-cleaning capability. In summary, this work demonstrates a novel strategy for designing multifunctional absorbing metamaterials, contributing to the development of advanced stealth materials.

## 2. Materials and Methods

### 2.1. Materials and Reagents

CIP was procured from Hengbei Metal Materials Co., Ltd., Hengbei, China. ER and Ultraviolet Rays (UV) resin were sourced from Wenzhou An’ergu New Materials Co., Ltd, Wenzhou, China. The anhydrous ethanol with the purity of 97% was obtained from Aladdin Biochemical Technology Co., Ltd, Shanghai, China. All materials were commercially available and utilized without additional processes.

### 2.2. HFSS Simulation Method

**Figure 1:**
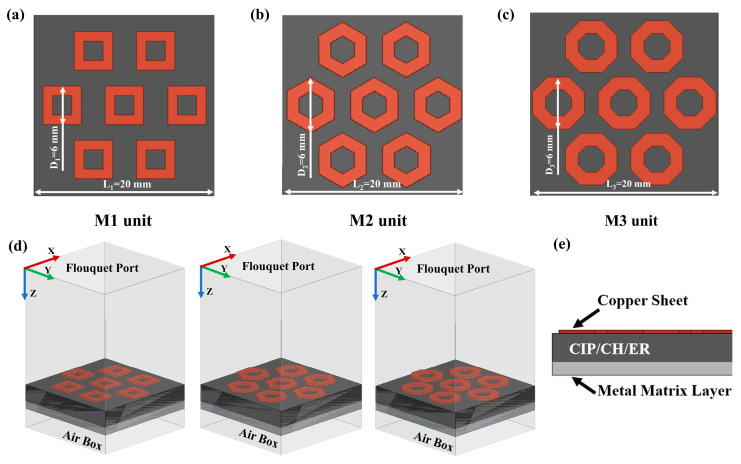
Images of (**a**) M1 unit, (**b**) M2 unit, and (**c**) M3 unit. (**d**) Schematic diagram of the simulation models for the M1, M2, and M3 units. (**e**) Images of the front view of the absorbing metamaterial.

The electromagnetic absorption performance of the metamaterial was evaluated over the 2–18 GHz range using Ansys HFSS 15 based on the finite element method. To simulate an infinite periodic array, a metamaterial unit was modeled with Master–Slave periodic boundary conditions and Floquet ports were utilized for excitation. The incident angle was set to 10° to align with the experimental arch method. To ensure the accuracy and stability of the numerical solution, adaptive mesh refinement was employed. The convergence criterion was defined by a maximum delta S of 0.02 with a limit of 15 adaptive passes, ensuring that the mesh was sufficiently refined in regions of electromagnetic fields. Furthermore, the complex permittivity and permeability of the CIP/CH composite were incorporated into the HFSS material library to analyze the absorption performance of the metamaterial coating (Figure 1).

### 2.3. The Manufacturing Procedure of Absorbing Metamaterial

**Figure 2:**
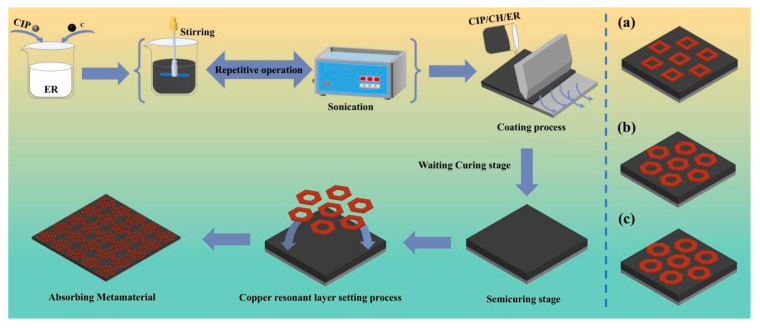
Fabrication and structure design of the absorbing metamaterials. Copper resonant layer of (**a**) square structural unit, (**b**) hexagonal structural unit, and (**c**) octagonal structural unit.

Initially, a 200 mm × 200 mm × 1 mm aluminum plate was selected as the metal substrate. Afterwards, it underwent ultrasonic cleaning in ethanol to remove surface contaminants. For the microwave absorbent preparation, CIP and CH powders were mixed at 5:X weight ratios (where X = 0.1, 0.15, 0.2, 0.25, 0.3) to achieve optimal electromagnetic properties. The mixture was dispersed into the ER matrix through mechanical stirring at 800 rpm for 30 min and subsequent sonication at 20 kHz for 30 min. This procedure was repeated to ensure a homogeneous distribution. It should be noted that ER was specifically selected because of its wave-transparent characteristics. The resulting CIP/CH/ER composite was then coated on the metal substrate. We positioned the copper sheets on the composite surface according to the designed array. The composite was then fully cured at room temperature for 6 h, resulting in a metamaterial (Figure 2).

### 2.4. The Preparation Process of Hydrophobic Coating

**Figure 3:**
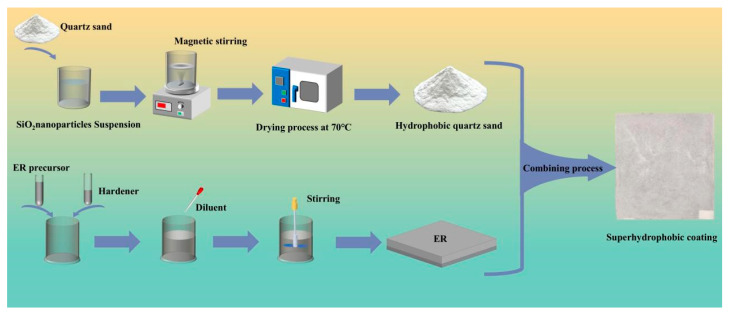
Fabrication of the superhydrophobic coating.

Silica nanoparticles were dispersed in anhydrous ethanol containing a silane coupling agent to prepare suspensions with different concentrations (0–0.015 g/mL). The suspension was then mixed with quartz sand under magnetic stirring. After drying at 70 °C, surface-modified hydrophobic quartz sand was obtained. The ER precursor and hardener were mixed at a weight ratio of 3:1 and stirred until a transparent mixture formed. A small amount of diluent was subsequently added to improve processability. During the initial stage of curing, the hydrophobic quartz sand was uniformly distributed over the resin surface. After complete curing, the final superhydrophobic coating was obtained, exhibiting both self-cleaning capability and mechanical durability. The coating was then attached to the metamaterial surface using UV resin as an adhesive to ensure firm bonding. As a result, the metamaterial retained its absorption performance while gaining stable self-cleaning functionality. This enabled the metamaterial to retain its absorption properties while acquiring stable self-cleaning functionality (Figure 3).

### 2.5. Materials Characterization

X-ray diffraction (XRD-Empyrean, Panalytical, Almelo, The Netherlands) was employed to determine the phase composition of CIP and CH powders, followed by data analysis using Jade 6.5 software. The BET surface area was measured by a high throughput specific surface and pore size analyzer (TriStar II Plus 3030, Micromeritics, Norcross, GA, USA). WCA measurements were performed with 2 µL water droplets using a goniometer (Powereach JC2000D1, Powereach, Beijing, China), while dynamic droplet adhesion was monitored using an CCD optical system (Olympus DP27, Powereach, China).

The surface morphology of CIP and CH power were characterized by field-emission scanning electron microscopy (Hitachi SU8010, Hitachi, Tokyo, Japan). The electromagnetic parameters of the CIP/CH composite were measured using a vector network analyzer (Agilent HP8722ES, Agilent, Santa Clara, CA, USA) via the coaxial reflection/transmission method. For measurement, the CIP/CH composite was mixed with paraffin and pressed into cylindrical samples (inner diameter: 3.04 mm; outer diameter: 7.00 mm). Paraffin is a wave-transparent matrix, with no impact on electromagnetic waves. The CIP/CH/paraffin composite (weight ratio: 6:0.24:1) achieved an absorbent volume ratio nearly identical to that of the CIP/CB/EP samples (weight ratio: 5:0.3:1), ensuring consistent electromagnetic absorption characteristics. The RL of the metamaterial was measured using the arch method and a vector network analyzer (Agilent HP8720B, Agilent, USA) at an incident angle of 10° over the 2–18 GHz.

## 3. Results

### 3.1. Microstructures of CIP/CH Composite Absorbent

**Figure 4:**
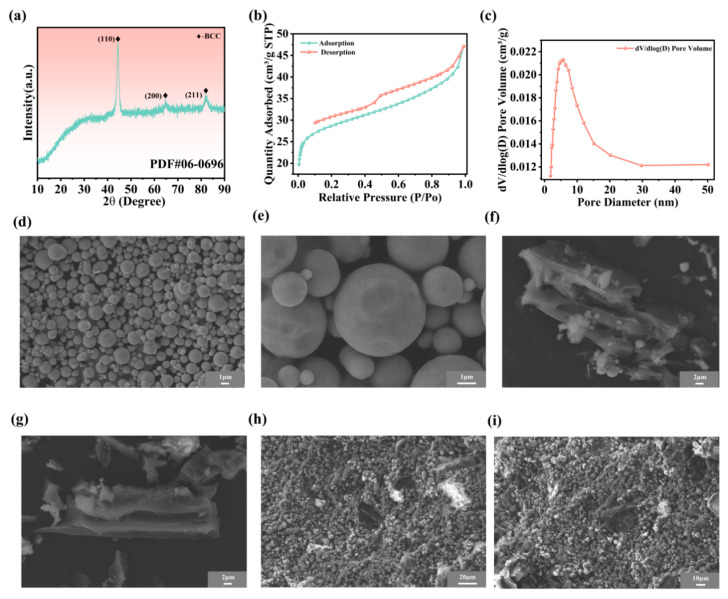
(**a**) The XRD pattern of CIP. (**b**) The nitrogen adsorption isotherms of CH samples. (**c**) The pore size distribution of CH samples (**d**,**e**) SEM images of the CIP. (**f**,**g**) SEM images of the CH powder. (**h**,**i**) SEM image of the fracture surface of the CIP/CB/ER composite.

The XRD pattern of CIP exhibits a typical body-centered cubic (bcc) structure, with characteristic diffraction peaks located at 2θ = 44.64° (110), 65.46° (200), and 82.29° (211), as shown in Figure 4a. As observed in Figure 4d,e, the CIP presents a spherical morphology with particle diameters ranging from 3 to 5 um, whereas the CH particles display irregular polygonal shapes in Figure 4f,g. The nitrogen adsorption isotherm and pore size distribution curves in Figure 4b,c confirm the mesoporous of CH.

The nanoscopic mesopores play a fundamental role in expanding the specific surface area of CH to 109.64 m^2^/g. This value represents a significant increase compared to the 0.32 m^2^/g observed for CIP. The high specific surface area and mesoporous structure of CH enable the construction of the heterogeneous interfaces even at low filler concentrations. These contact regions facilitate intense interfacial polarization, which achieves electromagnetic loss. As shown in Figure 4h,i, CH and CIP particles are uniformly dispersed within the composite without obvious aggregation. Such uniform dispersion contributes to stable electromagnetic parameters, which is beneficial for maintaining consistent wave transmission and reducing reflection and refraction losses.

### 3.2. Electromagnetic Characteristics of CIP/CH Composite Absorbant

The complex permittivity and permeability of materials affect the inherent absorbing performance, where the real parts (ε′, μ′) represent energy storage capacity and the imaginary parts (ε″, μ″) reflect dissipation loss [21,22,23,24]. In addition, tan δ (ε″/ε′) and tan μ (μ″/μ′) indicate the dielectric and magnetic loss mechanisms, respectively [25,26]. Figure S1 reveals that CH primarily enhances dielectric loss, while CIP contributes to magnetic loss. However, the high magnetic permeability of CIP or the high dielectric constant of CH will affect the absorption performance. Since the electromagnetic properties of composites are critically dependent on component ratios [27], we fabricated the CIP/CH composites with an optimized weight ratio to enhance absorption performance.

**Figure 5:**
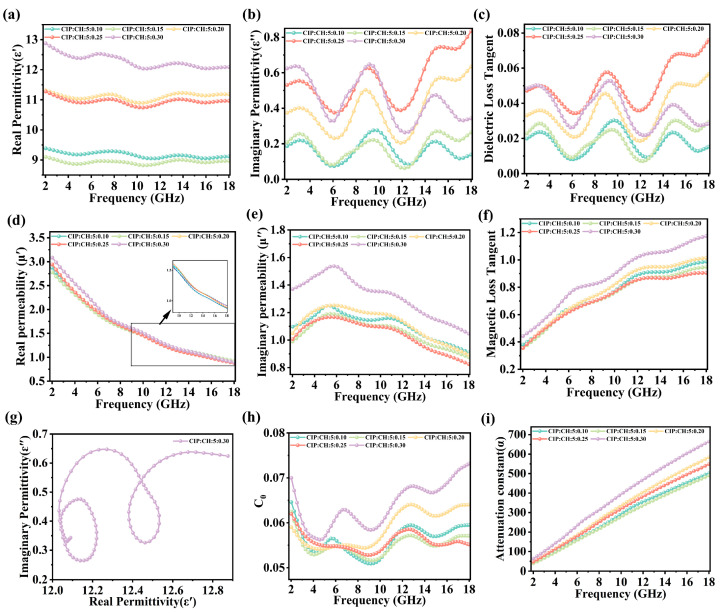
(**a**,**b**) Complex permittivity of CIP/CH composite. (**d**,**e**) Complex permeability of CIP/CH composite. (**c**,**f**) Dielectric and magnetic loss tangent of CIP/CH composite. (**g**) Cole–Cole curves of CIP/CH composite (weight ratios: 5:0.30). (**h**) C_0_ curves of CIP/CH composite. (**i**) Attenuation constant of CIP/CH composite with diverse weight ratio.

To clarify the electromagnetic wave absorption mechanism of CIP/CH composite materials, we first analyze the complex permittivity. As shown in Figure 5a, the real permittivity (ε′) of CIP/CH composites decreases with decreasing frequency due to dispersive properties [28,29,30]. The fluctuation of the imaginary permittivity (ε″) indicates that the composite possesses multiple polarization relaxation mechanisms [31,32]. These processes may stem from the dipole polarization in CH and the interface polarization generated by the contact between CH and CIP [33,34]. These mechanisms are further supported by the Cole–Cole plots (Figure 5g and Figure S2), where each semicircular arc corresponds to a distinct relaxation process [35,36]. Figure 4d demonstrates that the real permeability (μ′) of the CIP/CH composites exhibits a decline across the 2–18 GHz band. However, Figure 5e shows that the CIP/CH composites (weight ratio: 5:0.3) have superior magnetic loss properties due to their higher imaginary permeability (μ″). This suggests that an optimal CH content effectively enhances the magnetic loss of the composites. We further elucidated the underlying mechanism by plotting the C_0_ (C_0_ = μ″(μ′)^−2^f^−1^) [37,38] parameter of CIP/CH composites. As shown in Figure 5h, all CIP/CH composites exhibit the same fluctuating trend across 2–18 GHz, indicating that the magnetic loss is dominated by natural resonance and eddy current effects [39]. This consistency confirms that the magnetic loss mechanism is intrinsic to CIP and that no additional mechanism is introduced upon the addition of CH. Furthermore, it was found that the C_0_ values of the composite with a weight ratio of 5:0.3 are higher than those of a composite with a ratio of 5:0.1. This indicates that although CH itself possesses no inherent magnetism, the optimized weight ratio (5:0.3) enables a higher effective magnetic loss efficiency at a fixed CIP content.

Compared with the individual components (CIP or CH) that are limited to a single loss mechanism, the CIP/CH composite effectively integrates both dielectric and magnetic losses. A comparison of Figure 5c,f reveals a consistently higher tan μ (μ″/μ′) relative to tan δ (ε″/ε′) across the 2–18 GHz. Notably, magnetic loss is the dominant absorption mechanism of the CIP/CH composite. We then calculated the impedance matching (Zr) of CIP/CH composites at different weight ratios using the formula [40,41] (Equation (1)).

(1) $$Z_{r}=\frac{Z_{in}}{Z_{0}}=\sqrt{\frac{\varepsilon_{r}}{\mu_{r}}}\tanh\left(j\frac{2\pi fd}{c}\sqrt{\mu_{r}\varepsilon_{r}}\right)$$

where *Z_in_* denotes the absorber input impedance and *Z*_0_ denotes the free space impedance. Figure S3 presents the 2D distribution map of *Z_r_* for CIP/CH composites across tested mass ratios.

The green region denotes ideal impedance matching that of [42]. At a thickness of 2 mm, favorable impedance matching was achieved across both the C and X bands for CIP/CH composites with varying mass ratios. The CIP/CH composite with a weight ratio of 5:0.15 exhibited the optimal impedance matching, with *Z_r_* values approaching 1 across the 6–10 GHz range. With a further increase in thickness to 5 mm, the matching region of CIP/CH composite moves to the S band and reduces in area. This frequency shift aligns with the quarter-wavelength cancelation principle. In addition, the attenuation constant [43,44] (α) is employed to evaluate the microwave absorption performance of the material, calculated as follows

(2) $$\alpha =\frac{\sqrt{2}\pi f}{c}\times \sqrt{\left(\mu^{\prime \prime }\varepsilon^{\prime \prime }-\mu^{\prime }\varepsilon^{\prime }\right)+\sqrt{{\left(\mu^{\prime }\varepsilon^{\prime \prime }+\mu^{\prime \prime }\varepsilon^{\prime }\right)}^{2}+{\left(\mu^{\prime \prime }\varepsilon^{\prime \prime }-\mu^{\prime }\varepsilon^{\prime }\right)}^{2}}}$$

Figure 5i clearly shows that the α value of the CIP/CH composite material increases with increasing activated charcoal content. The high attenuation coefficient of the CIP/CH (5:0.3) composite is attributed according to Equation (2) to its concomitantly high ε″ and μ″. This result demonstrates that optimizing the composition of the CIP/CH composite is an effective method for enhancing the electromagnetic wave attenuation performance.

### 3.3. Electromagnetic Waves Absorbing Property of Metamaterial

**Figure 6:**
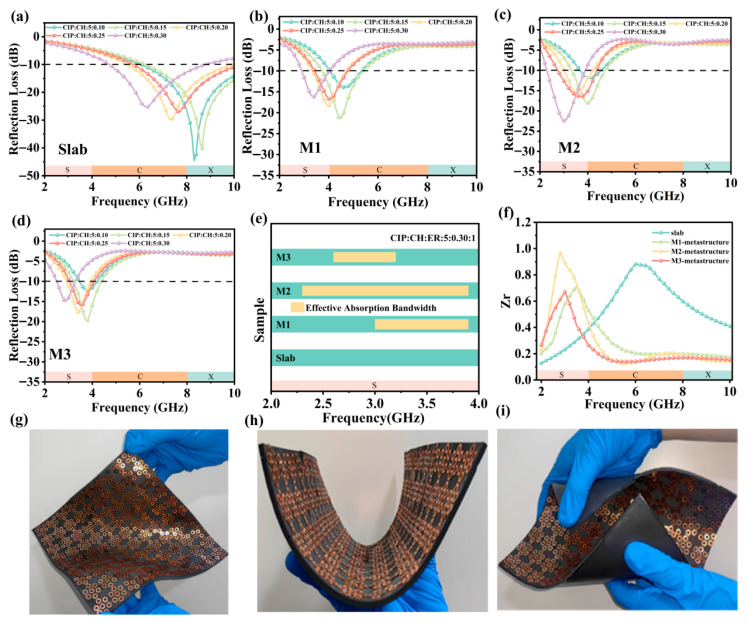
RL curves of (**a**) slab structures, (**b**) M1 structures, (**c**) M2 structures, and (**d**) M3 structures. (**e**) EAB of slab structures and three absorbing metamaterials structures (M1, M2, M3). (**f**) The Zr of different absorbing metamaterials and slab with CIP/CH/ER composite. (**g**–**i**) Flexible demonstration of M2 structure.

The absorption performance of a material is primarily evaluated by the RL and the effective absorption bandwidth [45]. The effective absorption bandwidth (EAB) is defined as the frequency range over which the RL is below −10 dB, indicating that more than 90% of the incident electromagnetic wave energy is absorbed [46]. Figure S4 shown the RL of CIP/CH composites with varying thicknesses over the 2−18 GHz frequency. The EAB and absorption peaks of the composite gradually toward the S-band (2–4 GHz) as the thickness increases. Although the 5 mm composite slab achieves effective S-band absorption, its mass conflicts with lightweight design requirements. In contrast, the 2 mm slab achieves broadband absorption in the C-X band (4–12 GHz) and also satisfies lightweight design. Figure 6a describes in detail the RL of the CIP/CH/ER slab structure at 2 mm thickness. The increase in CH content will shift the absorption peak frequency and EAB of composites from the X band (8–12 GHz) to the C band (4–8 GHz). Notably, we observe that the CIP/CH/ER composite slab (5:0.3:1 weight ratio) achieves a minimum RL (RLmin) of −25 dB at 6.3 GHz and an EAB of 4.7–8.7 GHz. While higher CH content extends the EAB toward lower frequencies, the CIP/CH composite still fails to achieve effective absorption (RL < −10 dB) in the S-band.

S-band absorption depends not only on material composition but critically on properly structural design. This study proposes combining a 0.25 mm metallic resonator layer with a 2 mm-thick CIP/CH/ER composite plate to design a novel metamaterial for achieving S-band absorption. Three metamaterials with square (M1), hexagonal (M2), and octagonal (M3) units arranged in nested hexagonal arrays were designed. We simulated their absorption performance using Ansys HFSS 15 and selected the optimal structure based on EAB and minimum RL. The RL of three absorbing metamaterial with designed metallic resonant layers (M1–M3) is displayed in Figure 6d–f. The results demonstrate that compared to the uniform slab, all those three metamaterials exhibit a shift toward lower frequency in both EAB and RLmin, primarily covering the S-band (2–4 GHz) and C-band (4–8 GHz). The M1 structure with quadrilateral units achieves EAB (RL < −10 dB) from 3.8 to 5.3 GHz and a minimum RL of −21 dB at 4.5 GHz through optimized CIP/CH/ER weight ratio. However, its EAB remains primarily confined to the C-band, failing to meet S-band broadband requirements. To address this limitation, we developed the M2 structure with hexagonal units. The M2 structure with CIP/CH/ER composites (weight ratio: 5:0.3:1) exhibits effective absorption (RL < −10 dB) in 2.3–3.9 GHz, achieving a RLmin of −27 dB at 3 GHz. In contrast, the M3 structure using octagonal cells performs worse than M2, with a maximum EAB of only 1 GHz and a RLmin of −19.6 dB at 3.8 GHz. Therefore, the M2 structure achieves broadband absorption (1.6 GHz) in the S band with a subwavelength thickness (d ≤ 3 mm). To further substantiate the advantages of the M2 metamaterial, a comparative analysis with recently reported S-band absorbers [13,47,48,49] is summarized in Table 1. While the compared absorbers exhibit effective absorption in the S-band, the M2 metamaterial demonstrates a superior balance between reduced thickness and enhanced bandwidth, confirming its efficacy for low-frequency applications.

This shows that the introduction of metal resonant layer will lead to peak shift and corresponding absorption band shift. These variations can be attributed to the changes in impedance and propagation path. Based on the equivalent circuit theory, the metamaterial is modeled as an equivalent RLC circuit, as shown in Figure S5. The metallic coppers function as inductive elements (L), while the gaps between adjacent units contribute to the equivalent capacitance (C). Based on the resonance condition $f=\frac{1}{2\pi \sqrt{LC}}$, the introduction of the resonator layer significantly increases the effective L and C values. This enhancement inherently lowers the resonance frequency, causing the absorption peak to migrate from the X-band toward the S-band. Such a shift confirms the effective tuning of the electromagnetic response via the structural design of the metal resonator layer.

Furthermore, the impedance-matching properties of the absorbing metamaterial are optimized through the metallic resonant layer of hexagon copper sheets at S band. Based on transmission line theory $RL=20\mathrm{lg}\left|\frac{Z_{r}-1}{Z_{r}+1}\right|$, the relative impedance (Z_r_) was derived to quantitatively evaluate the impact of the resonant layer on absorption performance. Utilizing this relationship, the calculated Z_r_ for the three metamaterials are depicted in Figure 6f. Specifically, the slab structure achieves the peak Z_r_ of only 0.8 at 6.2 GHz (C-band). In contrast, the introduction of the hexagonal copper unit (M2) significantly shifts the Z_r_ peak at toward the S-band (3 GHz) and enhances its maximum value to 0.9. This value is markedly closer to the ideal matching condition (Z_r_ = 1), and its peak frequency matches the minimum RL frequency. These quantitative comparisons confirm that the metal resonant layer design effectively optimizes impedance matching in the 2–4 GHz. Efficient microwave absorption necessitates a synergy between impedance matching and electromagnetic loss. The M2 structure achieves superior impedance optimization while simultaneously enhancing its loss capability through inter-unit electromagnetic coupling. These synergistic effects lead to the significantly improved S-band absorption performance.

The M2 metamaterial was fabricated and its RL was measured using the arch method to verify the feasibility of the structural design. As shown in Figure S6, the experimental RL curve agrees well with the simulated result, despite slight deviations. Specifically, the fabricated samples exhibit an experimental EAB of 1.7 GHz (2.8–4.5 GHz), which aligns with the simulated EAB of 1.5 GHz (2.3–3.8 GHz). In terms of absorption intensity, the measured RLmin reaches −23.6 dB, reflecting a minor deviation of 7.3% from the simulated value of −22.0 dB. Finally, the experimental peak frequency is observed at 3.2 GHz, representing a slight frequency shift of 6.7% relative to the simulated 3.0 GHz. These differences are mainly attributed to practical constraints in the measurement setup, particularly the limited distance between the antenna and the sample, whereas the HFSS simulation assumes ideal plane wave incidence. Despite these discrepancies, the experimental results confirm the feasibility of the M2 metamaterial. Traditional S-band metamaterial typically relies on rigid FR-4 substrates, which suffer from limited flexibility. In contrast, our proposed M2 metamaterial utilizes ER as a matrix to achieve exceptional flexibility. As demonstrated in Figure 6g–i, the material readily conforms to curved or irregular profiles. This metamaterial with a simplified fabrication process, ensures both economic viability and scalability for large-scale engineering applications.

### 3.4. Electromagnetic Responses Influenced by Structure

**Figure 7:**
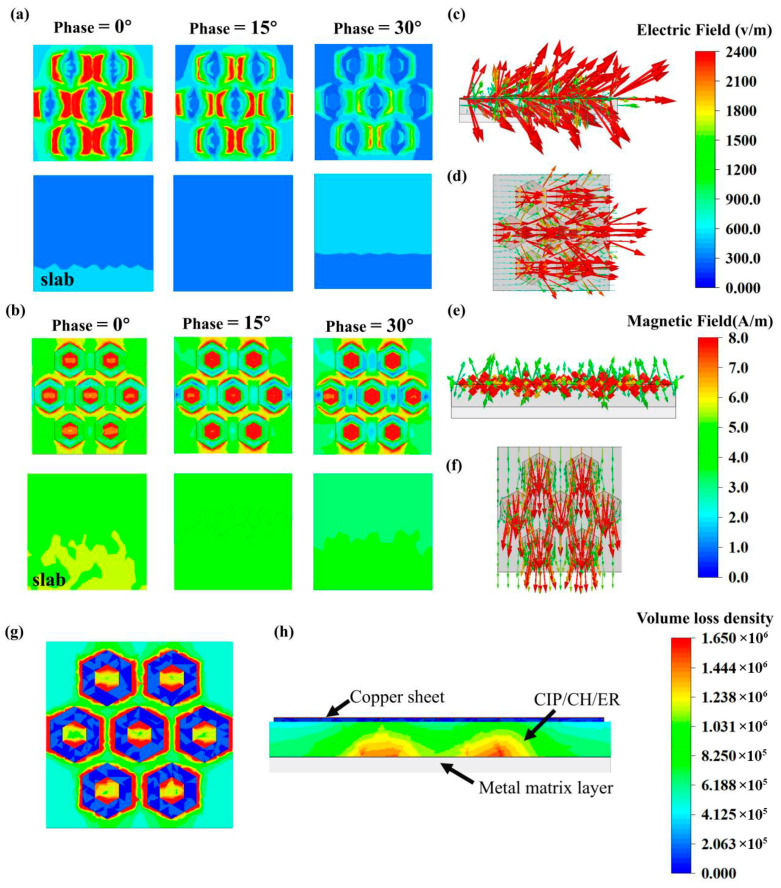
(**a**) Electric field distribution at 3 GHz of the M2-structured absorbing metamaterial and slab with various phases. (**b**) Magnetic distribution at 3 GHz of the M2-structured absorbing metamaterial and slab with various phases. Electric field vector distribution of the M2-structured absorbing metamaterial on (**c**) the front view and (**d**) the top view. Magnetic vector distribution of the M2-structured absorbing metamaterial on (**e**) the front view and (**f**) the top view. Volume loss density of the M2-structured absorbing metamaterial on (**g**) the top view and (**h**) the front view.

This study compares the electromagnetic field distribution and volumetric loss density of the slab and the M2 metamaterial at 3 GHz. As shown in Figure 7a, the electric field intensity of the M2 structure is significantly higher than that on the surface of the slab. While the slab primarily reflects incident microwaves at its surface—where reflected waves destructively interfere with incident ones, there reducing surface electric field intensity—the M2 structure employs a specialized metal resonant layer with a nested hexagonal array that effectively minimizes reflection. This mechanism enhances electromagnetic wave penetration into the metamaterial. Furthermore, the strong electric field concentrated at the edges of the resonant copper sheet induces edge diffraction and secondary scattering [50]. These mechanisms significantly improve the absorption performance of the M2 structure in the S-band.

The influence of the resonant layer on the magnetic field distribution was analyzed. As shown in Figure 7b, which illustrates the magnetic field distribution of the M2 structure and the slab at 3 GHz, a strong local magnetic field is observed at the edges of the copper sheet cells within the metal resonant layer. Notably, the field strength is maximized in the central regions of these copper structural units. This occurs because the incident electromagnetic waves induce a secondary magnetic field in the copper sheet, which combines with the primary field to produce a localized enhancement. This enhancement mechanism originates from the hexagonal array structure of the resonant layer. Specifically, this structure exploits its self-similarity to enhance the uniformity and intensity of the surface magnetic field, enabling stronger attenuation of S-band electromagnetic waves. Consequently, this design increases the local magnetic field strength and improves the uniformity of the field distribution, contributing to a superior attenuation capability in the S-band.

Figure 7c–f depicts the electric and magnetic field vector distributions, elucidating how the metal resonant layer enhances S-band absorption through local field enhancement and redistribution. Near the copper surface of the resonant layer, the electromagnetic field vectors exhibit an upward inclination. Notably, the field component perpendicular to the surface plays a pivotal role in these localized enhancement effects. By leveraging the orthogonality of electromagnetic waves, this vertical component facilitates a highly concentrated and redistributed electromagnetic field, thereby promoting stronger electromagnetic coupling in the metal resonant layer. Consequently, despite a significantly reduced thickness, the M2 superstructure absorptive coating maintains a performance comparable to that of a 5 mm-thick CIP/CH/ER homogeneous coating. This confirms that strategically designing the resonant layer to reshape the electromagnetic field distribution can effectively improve low-frequency absorption efficiency without increasing the material’s thickness. As shown in Figure 7g,h, the volumetric loss density calculations for the M2 structure reveal that the electromagnetic energy dissipation regions almost entirely overlap with the electric field concentration zones, indicating that the electromagnetic losses within the metal resonant layer primarily originate from electrical loss mechanisms.

This study elucidates the mechanisms by which metal resonant layers enhance S-band electromagnetic wave absorption. Electromagnetic simulations demonstrate that this specialized layer significantly amplifies the surface electric field intensity, minimizing surface reflection and facilitating deeper wave penetration in the metamaterial. Upon excitation by an external field, the copper plates within the resonant structure generate a secondary magnetic field, culminating in a localized field enhancement. By reshaping and intensifying the field vector distribution, this design enables the metamaterial to achieve effective S-band absorption at thickness substantially less than that of a conventional CIP/CH/ER slab. Furthermore, volumetric loss analysis confirms the dominance of electrical loss mechanisms in the M2 structure, as evidenced by the strong correlation between its electric field and volumetric loss density distributions. Ultimately, the nested hexagonal array of the metallic layer drives the electrical loss, while the CIP/CH/ER composite provides robust magnetic loss. The synergy of these mechanisms allows the metamaterial to achieve broadband absorption in the S-band at a subwavelength scale.

### 3.5. RCS Simulation Results

The RCS of the M2 metamaterial at 3 GHz was simulated to assess its stealth performance. The M2 metamaterial achieves optimum absorption performance at this frequency. The model is located in the x-y plane and is excited by a plane wave propagating along the −z direction. The scattering direction is defined by the spherical coordinates (θ φ).

**Figure 8:**
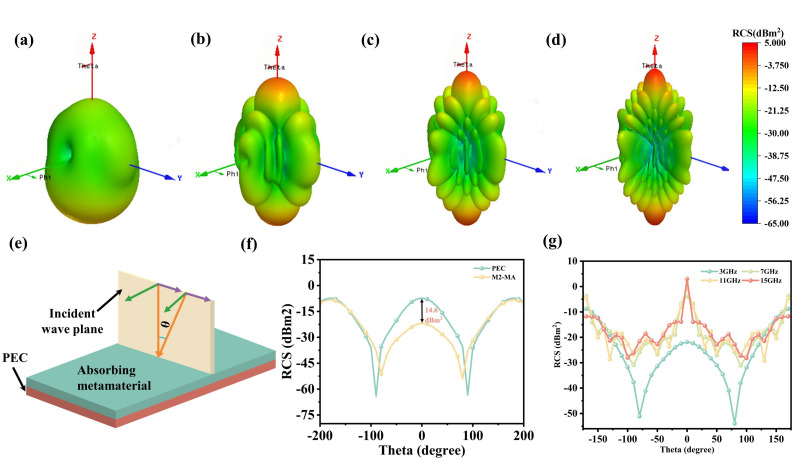
HFSS simulation three-dimensional RCS distributions of (**a**) the PEC with M2 coating at 3 GHz, (**b**) the PEC with M2coating at 7 GHz, (**c**) the PEC with M2 coating at 11 GHz, (**d**) the PEC with M2 coating at 15 GHz. (**e**) schematic diagram of RCS modeling in vertical polarization mode (**f**) The RCS values for PEC and M2 at different detection angles. (**g**) RCS values of M2 at different frequencies.

The PEC with M2 metamaterial coating demonstrates excellent radar stealth performance at a thickness of 2.25 mm. Specifically, the M2 metamaterial maintains an RCS below −10 dBm^2^ across a wide angular range of ±160°, as shown in Figure 8f. In addition, the 3D RCS distribution of the PEC coated with M2 in Figure 8a–d demonstrates effective radar-scattering reduction across multiple frequency bands. The M2 metamaterial achieves wide-angle stability and multi-frequency practicality by metal resonant layer and periodic array design. In summary, the M2 metamaterial presents a practical strategy for wide-angle RCS reduction that offers significant promise for stealth applications.

### 3.6. Metamaterial Hydrophobic Coating

**Figure 9:**
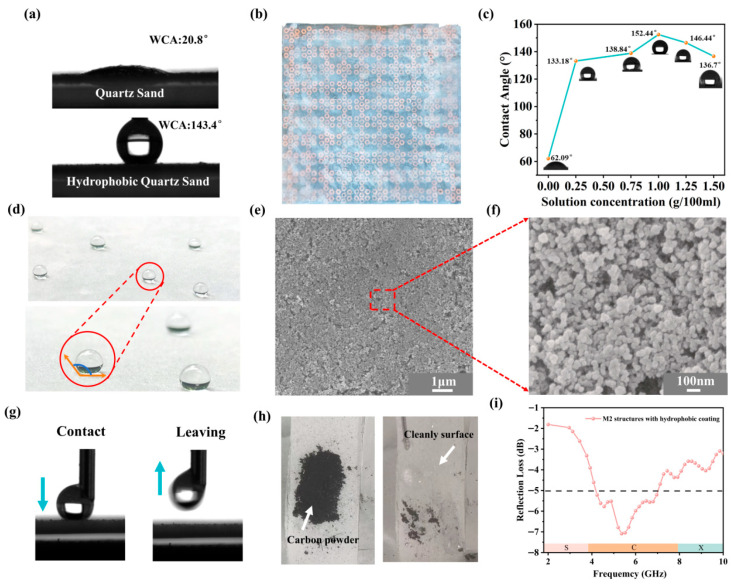
(**a**). The contact angles of quartz sand before and after modification treatment. (**b**) The M2 sample with hydrophobic coating. (**c**) The hydrophobic coating surface contact angles of various concentrations. (**d**) The schematic of water droplets on hydrophobic coating surfaces (1 g/100 mL). (**e**,**f**) SEM image of superhydrophobicity coating. (**g**) Images of water droplets following vertical movement on the surface of a superhydrophobicity coating. (**h**) Self-cleaning function of the superhydrophobicity coating. (**i**) The reflection loss of M2 with hydrophobic coating (1 g/100 mL).

The lotus leaves exhibit remarkable superhydrophobicity, attributed to their micro-nano structures and low-surface-energy wax layers [51,52]. A robust superhydrophobic coating was developed by grafting SiO_2_ nanoparticles onto quartz sand to provide essential self-cleaning capabilities for metamaterials in harsh environments. We used a SiO_2_ suspension with KH550 silane coupling agent to graft SiO_2_ particles on the surface of quartz sand [53]. The introduction of these nanomaterials proved to be the decisive factor for the performance leap. Following the optimization of the SiO_2_ concentration, the water contact angle increased from 20.8° to 143°, as shown in Figure 9a. These nanomaterials are essential for building the dual-scale hierarchical roughness that traps air pockets effectively, thereby minimizing the solid–liquid contact area. The hydrophobicity of quartz sand composite coatings was further improved by adjusting the SiO_2_ suspension concentration, as shown in Figure 9c. Specifically, the composite coating prepared from quartz sand treated with a 1 g/100 mL SiO_2_ suspension exhibited the WCA of 153°, exhibiting lotus-leaf-like superhydrophobicity. The performance originates from the micro-nano structures formed by quartz sand, which capture air to reduce the droplet contact area [54], as shown in Figure 9e,f.

However, when the SiO_2_ concentration was increased to 1.5 g/100 mL, the WCA decreased to 136.7°. This decline is attributed to agglomeration caused by excess SiO_2_ particle. The low adhesion of coating was confirmed by the easy lift of water droplets after complete contact with the surface, as shown in Figure 9g. Subsequently, carbon powder was spread to the coating surface as the pollutant and was easily removed by water rinsing, demonstrating excellent self-cleaning ability, as shown in Figure 9h. In summary, the prepared superhydrophobic coating effectively extends the service life of absorbing materials in harsh environments, facilitating their engineering application. As illustrated in Figure 9i, the M2 metamaterial maintains an effective electromagnetic wave absorption of approximately 68.4% in the C-band following the application of the hydrophobic coating. While prioritizing environmental durability incurs a minor penalty in absorption efficiency, achieving this optimal balance is justified for practical applications under complex environment.

## 4. Conclusions

Through compositional and structural optimization, we developed a multifunctional subwavelength metamaterial. The fabricated M2 metamaterial exhibits superior broadband absorption, covering most of the S-band (2.2–3.8 GHz) at a minimal thickness of 2.25 mm. This absorption performance arises from the combined effects of the effective loss mechanism introduced by the uniquely designed resonant layer and the intrinsic magnetic loss of the CIP/CH/ER composite. Electromagnetic simulations further reveal that the broadband S-band absorption of the metamaterial is enabled by the metal resonant layer, which concentrates the surface electric field and enhances the local magnetic field. In addition, the proposed structure exhibits an RCS below −10 dBm^2^ over an angular range of 160°. After the application of a quartz sand-based superhydrophobic coating (WCA = 152°), the M2 metamaterial acquires self-cleaning capability and improved long-term stability of its absorption performance. This work presents a subwavelength metamaterial integrating broadband S-band absorption with self-cleaning capabilities, offering a promising strategy for stealth applications. Since the current design is optimized for a 10° incidence, expanding its angular and polarization variations will be a key objective of our future research. Additionally, evaluating the mechanical durability and environmental stability of the structure will be the primary focus of our next research phase.

## Figures and Tables

**Table 1:** Performance comparison of absorbing metamaterials.

Absorber Type (Ref.)	Thickness (mm)	Absorption Domain (GHz)	EAB (GHz)
Split ring resonant structure [13]	9	1.24–3.14	1.6
Multiple layers resonant structure [47]	4.07	1.75–3.4	1.65
Triple-ring resonant structure [48]	3.585	2.3–4.3	2
Cross-shaped resonant structure [49]	2	2.5–5	2.5
M2 structure (This work)	2.25	2.3–3.9	1.6

## Data Availability

Data available on request from the corresponding author.

## Supplementary Materials

The following supporting information can be downloaded at https://www.mdpi.com/article/10.3390/nano16100620/s1.

## Abbreviations

The following abbreviations are used in this manuscript:

CIP	Carbonyl iron powder
CH	Charcoal
ER	Epoxy resin
RL	Reflection loss
EAB	Effective absorption bandwidth
RCS	Radar cross-section
WCA	Water contact angle
