# Supplementary material for: Ultrathin S-Band Multifunctional Metamaterial with Broadband Microwave Absorption and Hydrophobic Characteristics
Source: Nanomaterials (Basel). 2026 May 18;16(10):620. doi: 10.3390/nano16100620 (PMC13210102; doi:10.3390/nano16100620)
Supplement: Supplementary file 1 [file nanomaterials-16-00620-s001.zip › nanomaterials-4288315-supplementary.pdf]

Supporting Figures:

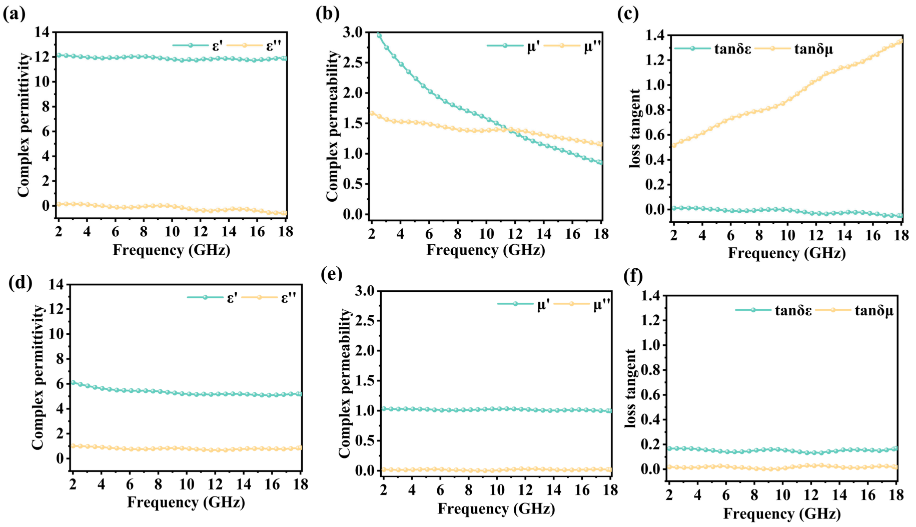

Figure. S1 Complex permittivity of (a) CH, (d) CIP. Complex permeability of (b) CH, (e) CIP. Dielectric and magnetic loss tangent of (c) CH, (f) CIP.

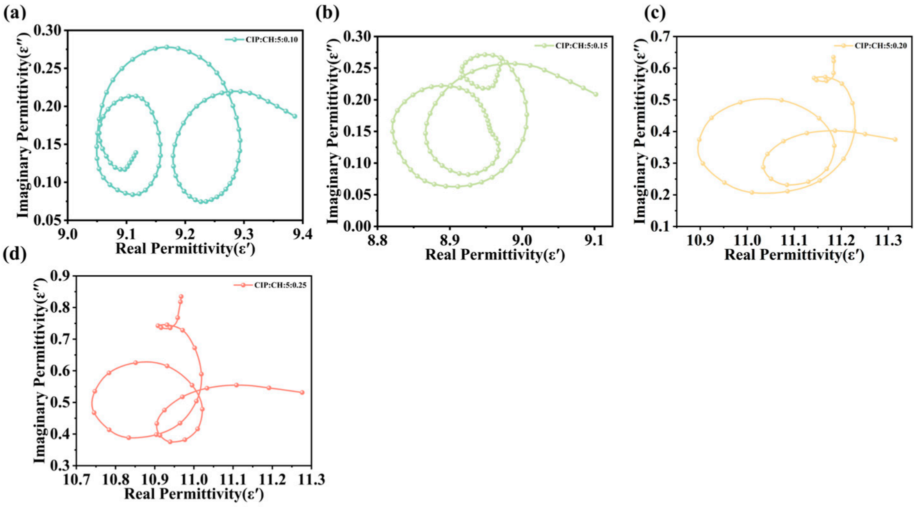

Figure. S2 Cole-Cole curves of (a) CIP/CH composite (weight ratios: 5:0.10), (b) CIP/CH composite (weight ratios: 5:0.15), (c) CIP/CH composite (weight ratios: 5:0.20), (d) CIP/CH composite (weight ratios: 5:0.25).

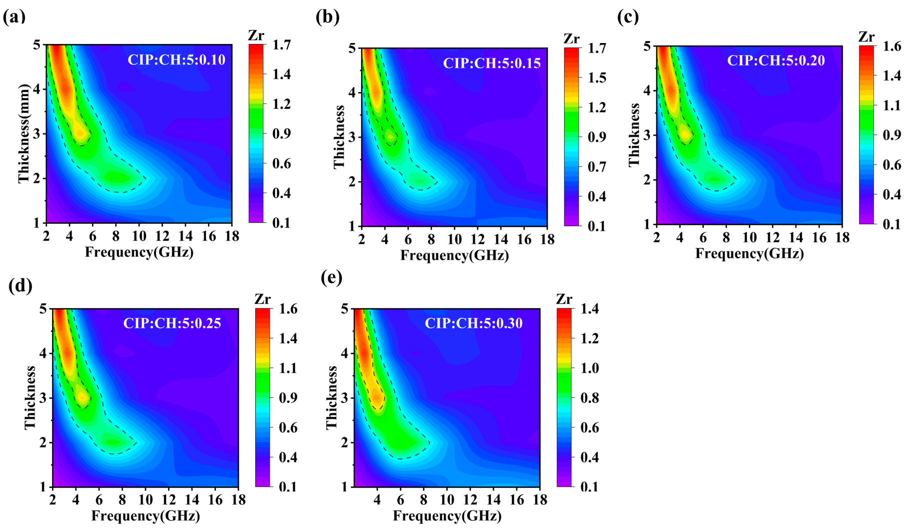

Figure. S3 The 2D distribution map of normalized impedance (Zr) of (a) CIP/CH composite (weight ratios: 5:0.10), (b) CIP/CH composite (weight ratios: 5:0.15), (c) CIP/CH composite (weight ratios: 5:0.20), (d) CIP/CH composite (weight ratios: 5:0.25), (e) CIP/CH composite (weight ratios: 5:0.30).

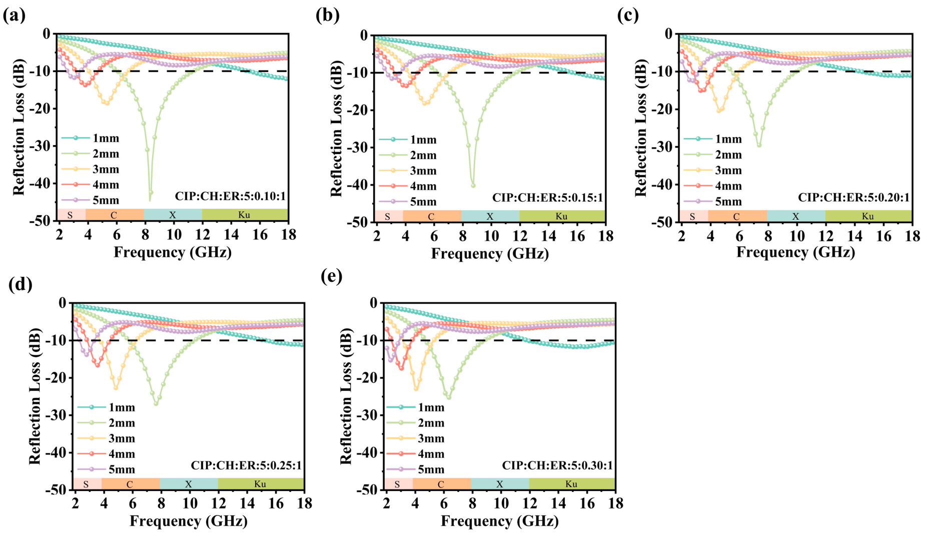

Figure. S4 The reflection loss with varying thickness of (a) CIP/CH composite (weight ratios: 5:0.10), (b) CIP/CH composite (weight ratios: 5:0.15), (c) CIP/CH composite (weight ratios: 5:0.20), (d) CIP/CH composite (weight ratios: 5:0.25), (e) CIP/CH composite (weight ratios: 5:0.30).

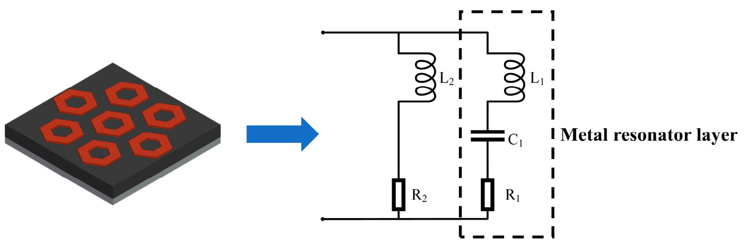

Figure. S5 The schematic diagram of the equivalent circuit in M2 metamaterial

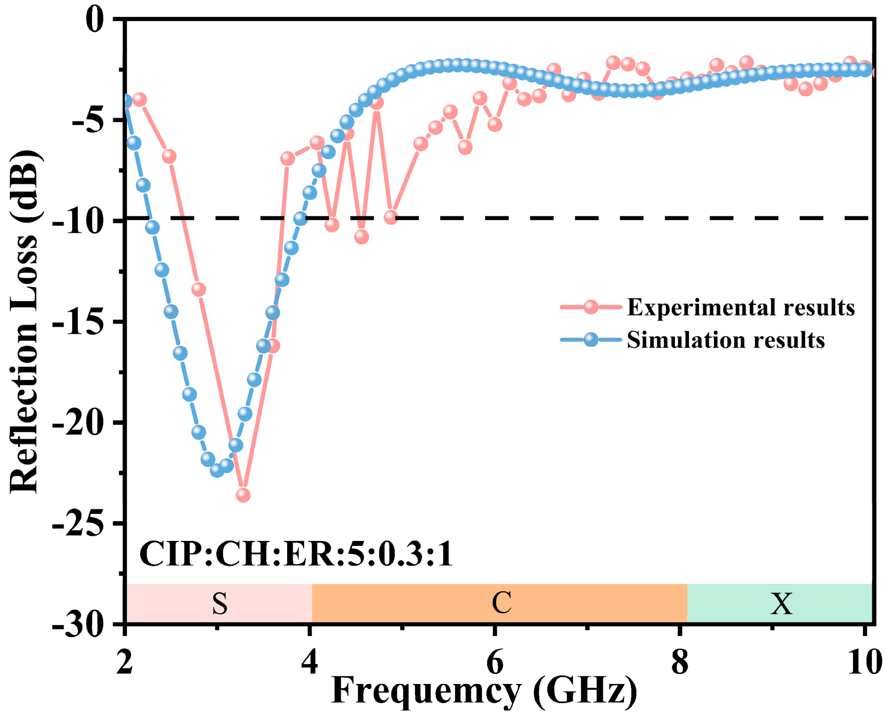

Figure. S6 Comparison of experimental and simulated results of the M2-structured metamaterial.

Supporting Videos:

Video S1. E field distribution in slab structure excited by phase-sweeping microwave at 3GHz
Video S2. H field vector distribution in M2 structure excited by phase-sweeping microwave at 3 GHz
Video S3. H field vector distribution in M2 structure excited by phase-sweeping microwave at 3 GHz
